# The relationship between under-five child death and maternal mental distress in Gamo Gofa Zone, Southern Ethiopia: a community based comparative cross-sectional study

**DOI:** 10.1186/s12905-018-0537-9

**Published:** 2018-02-27

**Authors:** Girma Temam Shifa, Ahmed Ali Ahmed, Alemayehu Worku Yalew

**Affiliations:** School of Public Health, Addis Ababa University, Addis Ababa, Ethiopia

**Keywords:** Mental distress, Common mental disorder, Maternal mental distress, Child loss, Child mortality, Gamo Gofa, Ethiopia

## Abstract

**Background:**

Knowledge of the association between child death and maternal mental distress may help to understand the indirect impact of reduction of under-five mortality on maternal mental wellbeing. This will further have a positive impact on the development of the nation. Depression is associated substantially with reduced quality of life and functional capacity of women. Although studies in the country assessed the magnitude of Common Mental Disorders (CMD) among postpartum mothers, those assessing the association between child death and maternal mental distress are lacking. Therefore, this study examined the association between child death and maternal mental distress.

**Methods:**

We conducted a comparative cross-sectional study in 2014 on a total of 356 mothers who lost their children and 712 mothers with alive children. We measured CMD symptoms using the World Health Organization’s (WHO’s) self-reporting questionnaire (SRQ-20). A cut-off score of ≥6 was taken as an indicator of mental distress. To determine the relationship between child death and maternal mental distress, we conducted weighted conditional logistic regression analysis with mental distress coded as a binary outcome.

**Results:**

Mothers who lost children had significantly higher rate of mental distress (adjusted odds ratio (AOR) of 1.84(1.11–3.04) compared to their counterparts. Similarly, mothers with child loss reported a significantly higher rate of suicidal ideation (23.3%) than mothers without child death (16.3%), with *p*-value of 0.003. The effect of child loss on maternal mental distress was greater during earlier periods (within 6 months of child death) and it decreased through time. However, it was shown to be persistently high at least during the first three years after child death, relative to mothers with alive child.

**Conclusions:**

Significantly higher proportions of women with child loss experienced mental distress including suicidal ideation than those without. Screening for maternal mental health problems by incorporating simple common mental distress assessing tools, like WHO’s SRQ into the maternal and child health care programs of health facilities may have significant effect on reducing the impact of maternal mental health problems in the designated communities.

## Background

In addition to economic losses mental disorders might bring, maternal mental disorder is associated with the wellbeing of children and the family. Studies have shown that early childhood failure to thrive (as measured by undernutrition and stunting), is significantly associated with depression in mothers [1, 2]. Maternal mental disorder was also shown to have negative effect on the subsequent pregnancy outcomes in the form of fatal congenital malformation, stillbirth and neonatal death [3]. Untreated maternal mental disorder has been shown to affect quality of child care [1] and child development [4, 5].

Depression in the mother may increase maternal mortality, through adversely affecting her physical health needs and through suicide. However, the earlier these conditions are recognized and addressed, the greater the chances of minimizing their impact [6].

Persons exposed to stressful life events are reported to experience mental disorders at a rate higher than those observed in the general population. For example, longitudinal studies of widowhood showed that bereaved ones may report psychological disturbance up to two years of the death of partner [7, 8].

The death of a child is, undoubtedly, a highly traumatic life event for parents. Such traumatic pressure is more notable for mothers [9, 10]. This may be because it is the mother who carry the child in her womb and directly feel the pain of labor. The rate of hospitalization for psychiatric illnesses was reported to be high among mothers with child loss than those without in a study which analyzed data from national registers in Denmark [9]. This may indicate that mothers with child loss are prone to even severe mental disorders that need hospitalization. In a study conducted in Australia, the death of a very young child, as in the case of stillbirth and neonatal death, has also been shown to invoke severe emotional distress in parents [10].

Another study in Australia also demonstrated that, the death of a baby has an impact on women’s mental health [11]. Mothers with child loss were found to be at increased risk for psychological distress at 2, 8, 15 and 30 months after their loss than mothers with live children [11]. A high rate of complicated grief among mothers who lost their children in health facilities was also reported by another study in United States of America [12] and complicated grief symptoms were shown to persist in some participants up to 18 months after child death by a subsequent study [13]. The negative effects of death of a child are also shown to be longstanding in a study which analyzed data from Wisconsin Longitudinal Study [14].

It has been shown, however, that brief counseling after such loss can significantly reduce morbidity in parents after child death [15]. There is also strong evidence that, all stressful life events do not invariably cause psychological disturbance. The susceptibility to stressful life events varies from person to person because of genetic factors, coping style, type of personality and social support the person may have [16]. Even following highly traumatic events, such as the death of a spouse, only minorities of individuals appear to develop serious or ongoing psychological disorder [7, 17]. The effects are expected to be context dependent that the situation in developing countries needs to be investigated as almost all the above studies are conducted in developed countries.

Showing the extent of the problem may help attract policy and programmatic directions of interventions. The recovery process is expected to be accelerated with appropriate psychiatric intervention that helps to settle an uncomplicated grief reaction [18]. Social support was shown to have better psychological outcomes among bereaved parents too [19]. Some social support issues identified as reducing symptoms of mental illness after bereavement comprise of: contact with other people, including friends, parents, spouses, surviving children, and other bereaved parents [20].

The assessment may also help to highlight the indirect impact of the reduction/prevention of child mortality on the maternal mental wellbeing which is shown to have a positive impact on the development of the nation as depression is associated substantially with reduced quality of life and functional capacity of the women [21]. It can also highlight the synergistic benefit of health interventions targeting either of the problems.

In Ethiopia, studies which assess the association between child death and maternal mental distress among bereaved mothers are lacking. Therefore, this study examined the association between child death and maternal mental distress. We hypothesized that mothers with child loss would have a higher rate of mental distress than mothers with live children.

## Methods

### Study area

This study was conducted in Gamo Gofa Zone. It has 15 Districts (Woredas) and two town administrations. Arba Minch Town, the Capital of the Zone, is 502 km south of Addis Ababa (the Capital of Ethiopia). Three hospitals and 68 health centers were providing health services for the population of the Zone during the study period. We purposefully selected Arba Minch Zuria District and Arba Minch Town for the study, out of the 15 Districts and two town administrations of the Zone, respectively. Arba Minch Zuria District, a home to Arba Minch Demographic Surveillance Site (DSS), had 29 kebeles (the smallest administrative units) located in different climatic zones (high land, mid land and low land). This is important to represent population of different agro ecological zones and thereby facilitating generalizability of the findings to different populations with similar backgrounds in Ethiopia in particular and in developing countries in general. As the Arba Minch DSS is a relatively young epidemiologic study site in the country, mental health studies are strictly limited. We included Arba Minch Town to represent the urban population of the Zone. Details of the study area were presented elsewhere [22].

### Study design and period

We conducted a comparative cross-sectional study in 2014.

### Source and study population

The source population was all mothers with under-five children in the study area. The study population consisted of exposed mothers, whose under five-children died in the period between April 01, 2011 and September 30, 2014 and un-exposed mothers with alive children, who were matched for date of birth of the index child (born within one month) in the same kebele. The duration was selected with the assumption that it enables us to have adequate sample without compromising the information (minimum recall bias).

### Inclusion and exclusion criteria

The study included only mothers who gave live-birth between September 01, 2007 and September 30, 2014 (i.e. still births were excluded). Those mothers who could not be located, communicated or died since then were excluded.

### Sample size determination

The sample size was determined using the statcalc program of Epi info 7 statistical software package. The prevalence of mental distress among mothers without child death (considered as unexposed) was estimated to be 19.8%, which was obtained from a study conducted among postnatal mothers in the country [23]. Then, the sample size required for detecting an odds ratio of 1.71 with a two-sided type one error of 5% and a power of 80% was determined with unexposed to exposed ratio of two. Accordingly, a minimum of 219 exposed and 438 unexposed (comparison group) were required. By applying a design effect of 1.5 and adding 10% to compensate for non-response, a total of 362 exposed and 724 unexposed mothers were required.

### Sampling technique

Purposefully, we selected the two districts (Arba Minch Zuria District and Arba Minch Town), out of the 15 Districts and two town administrations of the Zone, respectively. Then, we included all the 11 kebeles of Arba Minch Town and the nine kebeles of Arba Minch DSS. Initially, those kebeles of the Arba Minch DSS were randomly selected out of 29 kebeles of the District. Additionally, we selected 11 non-DSS kebeles of Arba Minch Zuria District randomly. Accordingly, we included 31 kebeles from the two Districts in the study. This part was explained in a relative depth elsewhere [22].

We conducted a census of all kebeles of Arba Minch Town and 11 non-DSS kebeles of Arba Minch Zuria District. Thereby, we identified all children (and their respective mothers) who were born between September 01, 2007-September 30, 2014 and under five children who died between April 01, 2011 and September 30, 2014. Additionally, as the Arba Minch DSS has been tracking all births and deaths since its establishment in 2009, we tracked children born between August 01, 2009 and September 30, 2014 and under five children who died between April 01, 2011 and September 30, 2014 in Arba Minch DSS kebeles from the database of the DSS.

Then, we classified all mothers who lost under-five children between April 01, 2011 and September 30, 2014 as exposed. For each exposed mother, we selected two mothers with alive children who were matched for date of birth of the index child (born within one month) and lived in the same kebele as a comparison group. After identifying all potential unexposed mothers for a specific exposed mother, we selected the two mothers randomly.

### Data collection

We measured common mental disorder (CMD) symptoms using the World Health Organization’s (WHO’s) self-reporting questionnaire (SRQ-20) [24]. This 20-item questionnaire asks about depressive, anxiety and somatic symptoms encountered in the past one month. The SRQ initially consisted of 25 questions (20 related to neurotic symptoms, 4 to psychotic symptoms and one asking about fits (convulsion)). Because of validation problems of SRQ-25, we used SRQ-20 for this study; a version validated in many countries including Ethiopia. This version of the instrument was used in previous Ethiopian community-based studies [25–28], and validated for use among perinatal women in Ethiopia [29]. A cut-off score of ≥6 was shown to have convergent validity [29]. We used the same cut-off point in this study. We employed interviewer administered method using the Amharic Version of the questionnaire. Besides, we utilized a quantitative questionnaire consisting of socioeconomic and demographic, health service utilization, reproductive characteristics of mothers.

### Measurements

#### Dependent variable

Presence of mental distress or not (on SRQ20, yes to greater than or equal to 6 of the 20 questions).

#### Independent variables

Primary: mortality status of the child. Secondary: socioeconomic &demographic, health service utilization, reproductive characteristics of the mothers.

### Operational definitions

#### Mothers/women having mental distress

Those women who answered ‘yes’ to six questions or more on the SRQ-20.

#### Wealth index

Was computed using principal component analysis (PCA) of reported ownership of household assets, such as radio, television, bicycle, livestock, etc.; and proxy indicators of living standard, including: number of rooms in the house, the roof of the house, whether the house was private or rental etc. The variables were adapted from the Ethiopian Demographic and Health survey (EDHS) questionnaire [30]. Then, the wealth status was categorized in to three groups and ranked from the poorest to the wealthiest.

#### Maternal power

Was also computed using PCA of eight variables that were adapted from EDHS 2011 [30], which measured decision power of the mother in the household (including: decision on maternal and husband income, on purchase and on visiting relatives or health facility (1 was assigned if the response favored maternal power (e.g. if mother decided alone or jointly with her partner) other wise 0); perception on wife beating (0 if she justified to at least for one of the options in the list); experience of ever being beaten by husband (0 if ever beaten) and if husband assisted in house chores (0 if husband didn’t assist)). Then, the maternal status was categorized into three groups and ranked from poor, average to good.

### Data processing, management and quality assurance

We pretested the study questionnaires in kebeles which were not included in the study. Three days training was given to data collectors and supervisors on the questionnaires and the procedures. The data collection process was strictly followed up and problems encountered were discussed and immediate solutions were sought. Besides, double entry of 10% of the questionnaires was made to identify any discrepancy. Supervisors and the principal investigator checked the collected data on daily basis for their completeness, clarity and consistency. Then, data were cleaned and checked before data entry and analysis again. The data were entered into computer using Epi Info Version 3.5.1.

### Data analysis

We performed, both descriptive and analytic analyses. We analyzed the data using SPSS Version 16 and STATA version 11 statistical packages, as required. The prevalence of mental distress among mothers who lost children within a specified time duration (e.g. within the past 6 months before the survey) was determined and graphed. We used open-epi version 2.3 to determine Extended Mantel-Haenszel chi-square for linear trend, in order to assess trends of level of mental distress among mothers with child loss as time went on. To determine the association between child death and maternal mental distress, we conducted weighted conditional logistic regression analysis with mental distress coded as a binary outcome (present or absent). The sampling weight was taken as the reciprocal of probabilities of selection. The probability of selection for urban was = 1/2*1*288/5745 = 0.025 [i) one out of 2 urban districts ii) all kebeles in the selected district were included and iii) 288 out of 5745 children identified by the census were included]. The corresponding sampling weight for urban was calculated to be 39.9. For that of rural =1/15*20/29*780/14416 = 0.0025 [i) one out of 15 rural districts, ii) twenty out of 29 kebeles of the district were included and iii) 780 out of 14,416 children identified were included in the study]. The corresponding sampling weight for rural was calculated to be 402.

We conducted model diagnostics using different STATA commands: For example, we assessed presence of specification error in the link term (logistic) or predictors with linktest command. Besides, we assessed the goodness of fit of the model by log likelihood chi-square test of the null hypothesis of no overall effect of predictors on the outcome and Akaike Information Criterion (AIC) to compare two models where one was nested on the other using estat ic command. We used collin command to assess presence of multicollinearity among predictors. Tolerance value of greater than or equal to 0.1 or variance inflation factor (VIF) of greater than or equal to 10 was taken as an indicator of presence of multicollinearity. All variables included in the model had VIF of less than five. We also assessed presence of interaction/effect modification for suspected variables in the model.

### Ethical considerations

The study obtained ethical clearance and approval from the Institutional Review Board of the College of Health Sciences at Addis Ababa University. Letters were written to all concerned bodies and we secured permissions at all levels. After explaining about the purpose of the study and confidentiality of the data, informed consent was obtained from each respondent. To assure confidentiality of the responses, anonymous interviews were conducted. Besides, the daily collected data were transferred to the Arba Minch University and locked in a secured cabinet.

## Results

### Socioeconomic-demographic characteristics of the respondents

We retrieved complete data from a total of 1068 mothers (356 exposed and 712 unexposed) giving a response rate of 98.3%. The overall mean (SD) age of the mothers was 27.1(6.2) years. Majority of exposed (87.4%) and unexposed (89.9%) mothers were in the age group of 18–35 years. Majority of exposed (75.0%) and unexposed (74.3%) mothers were from Gamo Ethnic Group. Most and comparable proportions of the exposed (67.4%) and unexposed (68.5%) mothers were Protestant Christians. More than half (56.2%) of the exposed mothers lacked formal education, whereas, less than half (46.9%) of the unexposed mothers had no formal education (Table 1).

**Table 1:** Socioeconomic-demographic characteristics of the participants, Gamo Gofa Zone, Ethiopia, 2014

Characteristics	Categories	Exposed	Unexposed	Total
		N (%)	N (%)	N (%)
Age category of the mother	< 18 yr	7(2.0)	11(1.5)	18(1.7)
	18-35 yr	311(87.4)	640(89.9)	951(89.0)
	> 35	38(10.7)	61(8.6)	99(9.3)
Ethnicity	Gamo	267(75.0)	529(74.3)	796(74.5)
	Gofa	7(2.0)	19(2.7)	26(2.4)
	Wolayta	25(7.0)	47(6.6)	72(6.7)
	Zeyse	37(10.4)	78(11.0)	115(10.8)
	Amhara	5(1.4)	19(2.7)	24(2.2)
	others	15(4.2)	20(2.8)	35(3.3)
Religion	Protestant	240(67.4)	488(68.5)	728(68.2)
	Orthodox	101(28.4)	196(27.5)	297(27.8)
	Others	15(4.3)	28(3.9)	43(4.1)
Educational level	No formal education	200(56.2)	334(46.9)	534(50.0)
	Grade1–6	100(28.1)	174(24.4)	274(25.7)
	Grade7–8	22(6.2)	71(10.0)	93(8.7)
	Grade9–12	18(5.1)	85(11.9)	103(9.6)
	Above grade12	16(4.5)	48(6.7)	64(6.0)
Occupation	Farmer	18(5.1)	35(4.9)	53(5.0)
	House wife	242(68.0)	503(70.6)	745(69.8)
	Gov’t employee	19(5.3)	48(6.7)	67(6.3)
	Merchant	41(11.5)	81(11.4)	122(11.4)
	Daily laborer	21(5.9)	11(1.5)	32(3.0)
	Housemaid	3(0.8)	4(0.6)	7(0.7)
	Student	9(2.5)	22(3.1)	31(2.9)
	Other	3(0.8)	8(1.1)	11(1.0)
Marital status	Married	326(91.6)	670(94.1)	996(93.3)
	Single	7(2.0)	20(2.8)	27(2.5)
	Divorced	6(1.7)	5(0.7)	11(1.0)
	Widowed	9(2.5)	4(0.6)	13(1.2)
	Separated	8(2.2)	13(1.8)	21(2.0)
Husband occupation	Farmer	177(54.3)	401(59.9)	578(58.0)
	Gov’t employee	32(9.8)	91(13.6)	123(12.3)
	Merchant	25(7.7)	64(9.6)	89(8.9)
	Student	5(1.5)	10(1.5)	15(1.5)
	Daily laborer	59(18.1)	53(7.9)	112(11.2)
	Other	28(8.6)	51(7.6)	79(7.9)
Husband education	No formal education	118(36.2)	208(31.1)	326(32.7)
	Grade 1–6	108(33.1)	187(27.9)	295(29.6)
	Grade 7–8	34(10.4)	78(11.6)	112(11.2)
	Grade 9–12	34(10.4)	113(16.9)	147(14.8)
	>Grade 12	32(9.8)	84(12.5)	116(11.6)
Wealth index	Poor	150(42.1)	278(39.0)	428(40.1)
	Average	90(25.3)	127(17.8)	217(20.3)
	Rich	116(32.6)	307(43.1)	423(39.6)
Maternal Power	Poor	124(34.8)	211(29.6)	335(31.4)
	Average	125(35.1)	260(36.5)	385(36.0)
	Good	107(30.1)	241(33.8)	348(32.6)

Majority of exposed (68.0%) and unexposed (70.6%) mothers were housewives. Majority of exposed (91.6%) and unexposed (94.1%) mothers were married at the time of the survey. Regarding husbands’ occupation,18.1% of exposed mothers’ husbands were daily laborers, while, only 7.9% of unexposed mothers’ husbands were daily laborers. Two hundred eight (63.8%) of exposed and 68.9% of unexposed mothers’ husbands had at least primary level education. Wealth index revealed that, 42.1% of exposed and 39% of unexposed mothers were from poor households. One hundred twenty-four (34.8%) of exposed and 29.6% of unexposed mothers had poor decision making status in the household (Table 1).

### Mental distress among the study participants

The internal reliability of the SRQ-20 was excellent with Cronbach’s alpha of 0.902. As depicted in Table 2, the most commonly reported symptoms included: “headache” (52.8%), “easily tire” (43.2%), “feel tired all the time” (40.2%) and “feel nervous, tense or worried” (38.2%). Overall, 18.6% of the mothers reported that they had suicidal ideation within the last 30 days before the survey. Significantly, higher rate of suicidal ideation was reported by exposed mothers (23.3%) compared to unexposed ones (16.3%), *p*-value of 0.003.

**Table 2:** Proportion of exposed and unexposed mothers who said yes to SRQ 20 questions, Gamo Gofa Zone, Ethiopia, 2014

S. No.	SRQ 20-Questions	Exposed (who said yes)	Unexposed (who said yes)	Total (who said yes)
		Freq. (%)	Freq. (%)	Freq. (%)
1.	Often had headache	201(56.5)	363(51.0)	564(52.8)
2.	Had poor appetite	132(37.1)	273(38.3)	405(37.9)
3.	Problem with sleep	90(25.3)	164(23.0)	254(23.8)
4.	Easily frightened	86(24.2)	161(22.6)	247(23.1)
5.	Hands shake	39(11.0)	66(9.3)	105(9.8)
6.	Feel nervous, tense or worried	159(44.7)	249(35.0)	408(38.2)
7.	Poor digestion	92(25.8)	153(21.5)	245(22.9)
8.	Trouble thinking clearly	108(30.3)	185(26.0)	293(27.4)
9.	Feel unhappy?	121(34.0)	189(26.5)	310(29.0)
10.	Cry more than usual	81(22.8)	114(16.0)	195(18.3)
11.	Difficulty to enjoy daily activities	106(29.8)	165(23.2)	271(25.4)
12.	Difficulty to make decisions	92(25.8)	147(20.6)	239(22.4)
13.	Daily work suffering	106(29.8)	174(24.4)	280(26.2)
14.	Unable to play a useful part in life	114(32.0)	188(26.4)	302(28.3)
15.	Lost interest in things	115(32.3)	175(24.6)	290(27.2)
16.	Feeling of worthlessness	111(31.2)	155(21.8)	266(24.9)
17.	Thought of ending life	83(23.3)	116(16.3)	199(18.6)
18.	Feel tired all the time	162(45.5)	267(37.5)	429(40.2)
19.	Uncomfortable feelings in stomach	116(32.6)	212(29.8)	328(30.7)
20.	Easily tired	164(46.1)	297(41.7)	461(43.2)
Positives for mental distress (95% CI)		48.0(42.9–53.2)	38.6 (35.1–42.3)	41.8 (39–45)

The overall mean (SD) score value (the number of yeses to the SRQ-20 questions) was 5.7 ± 5.1 ranging between 0 and 20. The mean (SD) scores for exposed mothers was higher than those of unexposed mothers (6.4 ± 5.3 vs 5.4 ± 5.1). Based on a cutoff point of six or above yeses to the SRQ questions, overall, 41.8% (95% confidence interval (CI) of 39%–45%) of the participants were positive for mental distress. The proportion of exposed mothers who were positive for mental distress was 48% (95% CI of 42.9%–53.2%), whereas only 38.6% (95% CI of 35.1%–42.3%) of unexposed mothers were positive (Table 2).

**Fig. 1:**
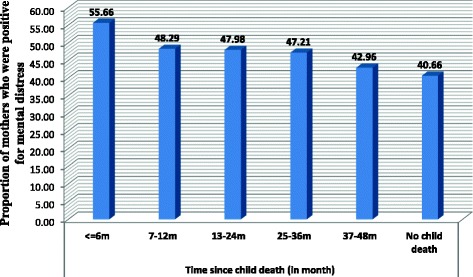
Proportion of mothers who had mental distress, by time since child death, Gamo Gofa Zone, Ethiopia, 2014

**Fig. 2:**
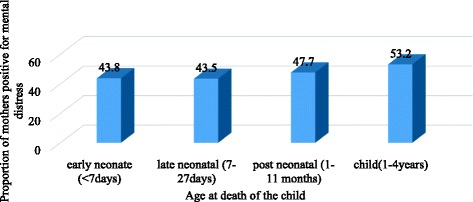
Proportion of mothers who had mental distress, by age of the child at death, Gamo Gofa Zone, Ethiopia, 2014

As shown in Fig. 1, the magnitude of maternal mental distress among mothers with child death was shown to be decreasing as time went on. The magnitude of mental distress was 55.7% among mothers who experienced child loss in the past six months. It decreased to 43% among mothers who experienced child loss more than three years before the survey, but it was persistently high among mothers with child loss than mothers without child loss at least for three to four years after child death (X^2^ = 10.65, *P* = 0.001) (Fig. 1). Maternal mental distress was also shown to be high if a child died at older age (Fig. 2).

### Association between child death and maternal mental distress

In the bivariate analysis, history of child loss was significantly associated with mental distress. The odds of mental distress was 1.6 times higher among mothers who lost their children than those who didn’t (crude odds ratio (COR) = 1.61(1.15–2.25)). After adjusting for potential confounding factors, the odds of mental distress among mothers with child death was about 1.8 times higher than among those mothers without child death (adjusted odds ratio (AOR) of 1.84(1.11–3.04) (Table 3).

**Table 3:** Independent association of maternal mental distress with child loss and other factors, Gamo Gofa, Ethiopia, 2014

Characteristics	Categories	COR	95% CI		AOR^a^	95% CI	
Exposure status	Un-exposed (mother with alive child)	Ref			Ref		
	Exposed (mother whose child is died)	1.61	1.15	2.25	1.84	1.11	3.04
Age of mother at birth		1.02	0.99	1.06	1.02	0.96	1.08
Ethnicity	Gamo	Ref			Ref		
	Zeyse	0.50	0.10	2.46	0.34	0.03	3.71
	Wolayta	2.22	0.72	6.88	3.28	0.92	11.70
	Others	0.98	0.31	3.95	0.88	0.27	2.87
Maternal education	No formal education	Ref			Ref		
	Grade1–6	1.16	0.67	1.99	***2.00***	***1.01***	***3.97***
	Grade7–8	0.94	0.43	2.06	2.17	0.88	5.36
	Above Grade9	0.70	0.32	1.53	1.86	0.66	5.30
Marital status	Married	Ref			Ref		
	Single	0.85	0.26	2.79	0.50	0.09	2.61
	Other	1.15	0.47	2.81	1.17	0.36	3.82
Mother Occupation	House wife	Ref			Ref		
	Farmer	0.49	0.21	1.15	0.68	0.26	1.73
	Gov’t employee	0.37	0.04	3.14	0.56	0.08	3.73
	Merchant	0.97	0.48	1.94	1.00	0.43	2.33
	Others	1.82	0.70	4.70	1.41	0.51	3.89
Wealth index	Poor	Ref			Ref		
	Average	***0.47***	***0.26***	***0.84***	***0.41***	***0.21***	***0.79***
	Rich	***0.39***	***0.20***	***0.77***	***0.31***	***0.14***	***0.67***
Maternal Power	Poor	Ref			Ref		
	Average	0.76	0.46	1.26	0.62	0.32	1.19
	Good	1.19	0.71	2.01	1.45	0.76	2.79
Ever beaten by her husband	Yes	Ref			Ref		
	No	0.98	0.53	1.82	1.08	0.49	2.40
Mother has any medical illness	Yes	Ref			Ref		
	No	***0.42***	***0.23***	***0.76***	***0.36***	***0.18***	***0.73***
Place of seeking health care	Traditional	Ref			Ref		
	Modern health service	***0.43***	***0.22***	***0.85***	***0.43***	***0.20***	***0.94***
Pregnant during survey	Yes	Ref			Ref		
	No	0.75	0.38	1.47	0.65	0.32	1.34
	Unsure	0.80	0.18	3.60	0.41	0.10	1.63
How many child has		1.02	0.93	1.12	1.09	0.93	1.28
Place of delivery	Home	Ref			Ref		
	Health post	0.98	0.36	2.63	0.52	0.17	1.61
	Higher health facility	0.87	0.47	1.60	0.58	0.25	1.37
Had Post-natal care during pregnancy of index child	Yes	Ref			Ref		
	No	1.00	0.58	1.73	0.54	0.25	1.18
Had ANC follow up during pregnancy of index child	Yes	Ref			Ref		
	No	***1.81***	***1.01***	***3.24***	***2.55***	***1.25***	***5.18***
Ever had child loss (including abortion & stillbirth) other than index child	Yes	Ref			Ref		
	No	***0.54***	***0.34***	***0.87***	0.60	0.33	1.08
Had live birth after index child	Yes	Ref			Ref		
	No	0.87	0.57	1.33	1.03	0.61	1.76

^a^Adjusted for all variables in the table & bold/italicized values are statistically significant values at 0.05

Other variables which were significantly associated with overall maternal mental distress included: wealth index of the household, whether the mother reported to have other medical illnesses or not, whether the family sought medical care from modern health system or not, whether the mother had antenatal care (ANC) follow up during the pregnancy of the index child or not and whether the mother had history of adverse pregnancy outcome (abortion or still birth) or child loss other than the death of the index child (Table 3).

With regard to wealth status, mothers from households of average (AOR of 0.41(0.21–0.79)) and rich (AOR of 0.31(0.14–0.67)) wealth indices had less odds of mental distress than those from poor categories. The odds of mental distress among mothers who did not report to have other medical problems (including heart problems, hypertension, diabetes mellitus etc.) was 64% (AOR of 0.36(0.18–0.73)) less than among those who reported to have any other medical problems. Similarly, odds of mental distress among mothers who reported seeking medical care from modern health facilities was 57% (AOR of 0.43(0.20–0.94)) less than among those mothers who reported to seek from traditional means (at home or traditional healers). Compared to those mothers who reported to have ANC follow up during pregnancy of the index child, the odds of mental distress was about 2.6 times (AOR of 2.55(1.25–5.18)) higher among those who did not have ANC follow up (Table 3).

## Discussion

Earlier studies in Ethiopia assessed the magnitude of mental distress among postpartum mothers [23] and the effect of maternal mental distress on child wellbeing [31, 32]. However, none of them tried to assess the association between child death and maternal mental distress. This study bridged this gap by exploring the association between child loss and maternal mental distress.

This study demonstrated that child loss is significantly associated with maternal mental distress. Mothers who lost their child had significantly high rate of mental distress than their counterparts. Similarly, mothers who suffered child loss reported a significantly high rate of suicidal ideation than mothers without child death. This finding is consistent with other previous studies [10, 11]. Similarly, high rate of hospitalization for psychiatric illnesses among mothers with child loss than those without was reported by another study [9]. This may be an indication that mothers with child loss are prone to even severe mental illnesses which may require hospitalization. These findings may imply the importance of integration of mental health interventions into other maternal health services in order to respond to the needs for mothers with child loss.

The effect of child loss on maternal mental distress was greater during earlier periods (within 6 months of child death) and it decreased through time. However, it was shown to be persistently high at least during the first three years after child death among mothers with child loss than their unexposed counterparts. Similar finding, persistent mental distress among mothers with child loss, was observed by other studies [11, 13]. While, mental distress was reported to persist up to 30 months after child death [11], risk of hospitalization for any psychiatric disorder was also reported to remain significantly high among mothers who lost their children even after five years of child death [9]. This persistent maternal mental distress after child loss may have an impact on the wellbeing of subsequent children, leading to a vicious cycle of the problem. Previous studies revealed a significant association between history of death of older sibling of the child with the death of the index child [33–35]. It was also shown that child wellbeing was significantly associated with maternal mental distress [1, 2, 31]. All those indicate the importance of providing mental health interventions targeting mothers with child loss, for the benefit of both mothers and their children.

Congruent of that, WHO recognizes the high rate of mental health problems during pregnancy and postnatal period, yet also the need for specialized support and psychotropic medicines only by small proportion of affected women. It therefore, recommends, training of health care providers at primary level to identify symptoms and signs suggestive of mental health problems and provide counselling to women about stress as well as other supportive interventions [6].

This study also assessed other factors associated with mental distress among all mothers. Overall, mothers from households of average and rich wealth index had less odds of mental distress than those from poor categories. Similar findings were reported by other studies [36, 37] indicating that poor women are more likely to have postpartum depression than rich ones. This may be related to stresses, worries and feelings of insecurities in feeding and sheltering their families or due to dissatisfactions in their current living situations.

Mothers who reported to have other medical problems had a high rate of mental distress than those without. Similar findings were reported by a study among postnatal mothers [36], which demonstrated that presence of other medical illnesses was significantly associated with mental distress. Another study also reported the same among the general population [38]. Bidirectional link between other medical illnesses and mood disorders was also underscored by a scientific review of literatures [39]. It was also reported that mood disorders affect the course of medical illnesses [39]. All these may signify the importance of integrating mental health interventions (including screening, counseling and referral of those in need) with management of other medical problems.

Mothers who had ANC follow up during their pregnancy of the index child had less odds of mental distress than those who did not. This may be because of the benefit of the general counseling provided by health workers on the general health of the mother and her child. This may be an indication for the benefit that mothers can gain if mental health related counseling is integrated with maternal health services, including during ANC follow up. Such counselling may help mothers to learn the health status of themselves and their coming newborn and cope with stressful events that they may face.

Because of the observational nature of the study design, some systematic errors, such as recall and social desirability biases could not be completely avoided. For example, the response of the respondents about household assets, mothers’ decision power in the household could be prone to recall and social desirability biases. Again, because of the cross-sectional nature of the study design, temporal relationship between child death and maternal mental distress could not be established in this study.

## Conclusions

In conclusion, high rate of mental distress prevails among women with child loss than those without. Significantly, high rate of suicidal ideation was also reported among affected mothers. Even though mental distress was shown to decrease through time, it persisted significantly high for relatively longer duration (at least three years after child death) among mothers who suffered child loss than those without. Those findings warrant the need to consider maternal mental health related interventions alongside those aiming to mitigate child mortality and improve mothers’ and their children’s wellbeing.

In this study, mothers who lacked ANC follow up during pregnancy of the index child were shown to have high rate of mental distress than those who had. Screening for maternal mental health problems by incorporating simple common mental distress assessing tools such as SRQ-20 into the maternal and child health care programs of health facilities (for screening, counseling and referral of those in need) may have significant impact in mitigating the impact of maternal mental health problems. As mothers with other medical illnesses were more prone to have high rate of mental distress, creating awareness of health service providers may help to reduce maternal mental health problems by enhancing appropriate intervention.

Beyond health system specific measures, as significantly high rate of mental distress was observed among mothers from poor households, improving the socioeconomic status of communities may be considered as one of the measures to prevent maternal mental health problems.

The feasibility and effectiveness of provision of simple psychosocial interventions, such as screening for maternal mental distress using simple screening tools, basic counseling and emotional support by front line health workers, such as health extension workers need to be explored in Ethiopia. Further study with stronger design will be helpful to establish cause-effect relationship between child death and maternal mental distress in Ethiopian context. Finally, as this study was conducted in areas similar; not only to most of the rural communities in Ethiopia but also to many other communities in developing countries in terms of demographics, health services and economic structure, its findings can be generalized to wider communities to inform context-specific interventions.

## Abbreviations

ANC: Antenatal care
AOR: Adjusted Odds Ratio
CI: Confidence interval
CMD: Common Mental Disorders
COR: Crude odds ratio
DSS: Demographic surveillance site
EDHS: Ethiopian Demographic and Health survey
PCA: Principal component analysis
SD: Standard deviation
SRQ: Self-reporting questionnaire
VIF: Variance inflation factor
WHO: World health organization

## References

[1] Patel V, Rahman A, Jacob KS, Hughes M (2004). Effect of maternal mental health on infant growth in low income countries: new evidence from South Asia. BMJ.

[2] Rahman A, Lovel H, Bunn J, Iqbal Z, Harrington R (2003). Mothers mental health & infant growth: a case-control study from Rawalpindi, Pakistan. Child Care Health Dev.

[3] King-Hele S, Webb RT, Mortensen PB, Appleby L, Pickles A, Abel KM (2009). Risk of stillbirth and neonatal death linked with maternal mental illness: a national cohort study. Arch Dis Child Fetal Neonatal Ed.

[4] Walker SP, Wachs TD, Gardner JM, Lozoff B, Wasserman GA (2007). Child development: risk factors for adverse outcomes in developing countries. Lancet.

[5] Cooper PJ, Murray L, Wilson A, Romaniuk H (2003). Controlled trial of the short- and long-term effect of psychological treatment of post-partum depression. Brit J Psychiat.

[6] World Health organization, Department of Mental Health and Substance Abuse. Improving Maternal Mental Health. Geneva. 2008. URL: http://www.who.int/mental_health/prevention/suicide/Perinatal_depression_mmh_final.pdf. Accessed 20 July 2015.

[7] Zisook S, Shuchter SR, Sledge PA, Paulus MP, Judd LL (1994). The spectrum of depressive phenomena after spousal bereavement. J Clin Psyehiat.

[8] Stroebe W, Stroebe MS, Domittner G (1988). Individual and situational differences in recovery from bereavement: a risk group identified. J Soc Iss.

[9] Li J, Laursen TM, Precht DH, Olsen J, Mortensen PB. Hospitalization for mental illness among parents after the death of a child. N Engl J Med 2005; 352:1190–1196. URL: http://www.nejm.org/doi/pdf/10.1056/NEJMoa033160. Accessed 15 July 2015. 10.1056/NEJMoa033160 15788495

[10] Vance JC, Foster WJ, Najman JM, Embelton G, Thearle MJ, Hodgen FM (1991). Early parental responses to sudden infant death, stillbirth or neonatal death. Med J Aust.

[11] Boyle FM, Vance JC, Najman JM, Thearle MJ (1996). The mental health impact of stillbirth, neonatal death or sudden infant death syndrome(SIDS): prevalence and patterns of distress among mothers. Soc Sci Med.

[12] Meert KL, Donaldson AE, Newth CJ, Harrison R, Berger J, Zimmerman J, Anand KJ, Carcillo J, Dean JM, Willson DF (2010). Complicated grief and associated risk factors among parents following a child's death in the pediatric intensive care unit. Archives of pediatrics & adolescent medicine.

[13] Meert KL, Shear K, Newth CJ, Harrison R, Berger J, Zimmerman J, Anand KJ, Carcillo J, Donaldson AE, Dean JM (2011). Follow-up study of complicated grief among parents eighteen months after a child's death in the pediatric intensive care unit. J Palliat Med.

[14] Rogers CH: The Effect of the Death of a Child on Midlife Mental and Physical Health: An Exploration of Risk and Resilience Factors". Psychology Dissertations. Paper 8. Georgia State University. URL: http://scholarworks.gsu.edu/cgi/viewcontent.cgi?article=1007&context=psych_diss. Accessed 10 Aug 2015, 2005.

[15] Forrest GC, Standish E, Baum JD (1982). Support after perinatal death: a study of support and counselling after perinatal bereavement. BMJ.

[16] Salleh MR (2008). Life event, stress and illness. Malays J Med Sci.

[17] Vachon MLS, Rogers J, Lyall WAL, Lancee WJ, Sheldon AR, Freeman SJJ (1982). Predictors and correlates of adaptation to conjugal bereavement. Am J Psychiatr.

[18] Lindemann E (1944). Symptomatology and management of acute grief. Am J Psychiatr.

[19] World Health Organisation-Regional Office for Europe. Impact of economic crises on mental health. Copenhagen, Denmark. 2007. URL: http://www.euro.who.int/__data/assets/pdf_file/0008/134999/e94837.pdf. Accessed 25 May 2015.

[20] Wheeler I (2001). Parental bereavement: the crisis of meaning. Death Studies.

[21] UNFPA/WHO. Maternal mental health and child health and development in resource-constrained settings: Report of a UNFPA/WHO international expert meeting: the interface between reproductive health and mental health, Hanoi, June 21–23, 2007. URL: http://apps.who.int/iris/bitstream/10665/70118/1/WHO_RHR_09.24_eng.pdf. Accessed 12 July 2015.

[22] Shifa GT, Ahmed AA, Yalew AW (2016). Early days of life are crucial for child survival in Gamo Gofa zone, southern Ethiopia: a community based study. BMC Pediatr.

[23] Baumgartner JN, Parcesepe A, Mekuria YG, Abitew DB, Gebeyehu W, Okello F, Shattuck D (2014). Maternal mental health in Amhara region, Ethiopia: a cross-sectional survey. Global health, science and practice.

[24] Beusenberg M, Orley J. World Health Organisation; A user's guide to the Self-Reporting Questionnaire (SRQ). 1994, Geneva. URL: http://apps.who.int/iris/bitstream/10665/61113/1/WHO_MNH_PSF_94.8.pdf. Accessed 10 Jan 2014.

[25] Alem A, Kebede D, Woldesemiat G, Jacobsson L, Kullgren G (1999). The prevalence and socio-demographic correlates of mental distress in Butajira, Ethiopia. Acta Psychiatr Scand.

[26] Senturk V, Hanlon C, Medhin G, Dewey M, Araya M, Alem A, Prince M, Stewart R (2012). Impact of perinatal somatic and common mental disorder symptoms on functioning in Ethiopian women: the P-MaMiE population-based cohort study. J Affect Disord.

[27] Damena T, Mossie A, Tesfaye M (2011). Khat chewing and mental distress: a community based study, in Jimma City, southwestern Ethiopia. Ethiop J Health Sci.

[28] Gelaye B, Lemma S, Deyassa N, Bahretibeb Y, Tesfaye M, Berhane Y, Williams MA (2012). Prevalence and correlates of mental distress among working adults in Ethiopia. Clin Pract Epidemiol Ment Health.

[29] Hanlon C, Medhin G, Alem A, Araya M, Abdulahi A, Hughes M, Tesfaye M, Wondimagegn D, Patel V, Prince M (2008). Detecting perinatal common mental disorders in Ethiopia: validation of the self-reporting questionnaire and Edinburgh postnatal depression scale. J Affect Disord.

[30] Ethiopia Demographic and Health Survey: Addis Ababa, Ethiopia and Calverton, Maryland, USA: Central Statistics Agency and ORC Macro 2011. 2011. https://dhsprogram.com/pubs/pdf/fr255/fr255.pdf. Accessed 15 Jan 2014.

[31] Ross J, Hanlon C, Medhin G, Alem A, Tesfaye F, Worku B, Dewey M, Patel V, Prince M (2011). Perinatal mental distress and infant morbidity in Ethiopia: a cohort study. Arch Dis Child Fetal Neonatal Ed.

[32] Medhin G, Hanlon C, Dewey M, Alem A, Tesfaye F, Lakew Z, Worku B, Aray M, Abdulahi A, Tomlinson M (2010). The effect of maternal common mental disorders on infant undernutrition in Butajira, Ethiopia: the P-MaMiE study. BMC Psychiatry.

[33] Kwakye KO, Otupiri E, Dabo EO, Browne ENL, Adjuik M (2010). Determinants of under-five mortality in Builsa District, upper east region, Ghana. J Sci Technol.

[34] Abir T, Agho KE, Page AN, Milton AH, Dibley MJ (2015). Risk factors for under-5 mortality: evidence from Bangladesh demographic and health survey, 2004-2011. BMJ Open.

[35] Kyei KA (2012). Determinants of childhood mortality in South Africa: using categorical data modeling. J Hum Ecol.

[36] Pollock JI, Manaseki-Holland S, Patel V (2009). Depression in Mongolian women over the first 2 months after childbirth: prevalence and risk factors. J Affect Disord.

[37] Chandran M, Tharyan P, Muliyil J, Abraham S (2002). Post-partum depression in a cohort of women from a rural area of Tamil Nadu, India. Incidence and risk factors. Br J Psychiatry J Ment Sci.

[38] Patel V, Kirkwood BR, Pednekar S, Weiss H, Mabey D (2006). Risk factors for common mental disorders in women. Population-based longitudinal study. Br J Psychiatry J Ment Sci.

[39] Evans DL, Charney DS, Lewis L, Golden RN, Gorman JM, Krishnan KR, Nemeroff CB, Bremner JD, Carney RM, Coyne JC (2005). Mood disorders in the medically ill: scientific review and recommendations. Biol Psychiatry.

